# Trends in health service use among persons with Parkinson’s disease by rurality: A population-based repeated cross-sectional study

**DOI:** 10.1371/journal.pone.0285585

**Published:** 2023-05-19

**Authors:** Laura C. Maclagan, Connie Marras, Isabella J. Sewell, C. Fangyun Wu, Debra A. Butt, Karen Tu, Susan E. Bronskill

**Affiliations:** 1 ICES, Toronto, Ontario, Canada; 2 Edmond J Safra Program in Parkinson Disease, Toronto Western Hospital, Toronto, Ontario, Canada; 3 Sunnybrook Health Sciences Centre, Sunnybrook Research Institute, Toronto, Ontario, Canada; 4 Department of Family and Community Medicine, Scarborough Health Network, Scarborough General Hospital, Toronto, Ontario, Canada; 5 Department of Family and Community Medicine, University of Toronto, Toronto, Ontario, Canada; 6 Management & Evaluation, Dalla Lana School of Public Health, Institute of Health Policy, University of Toronto, Toronto, Ontario, Canada; 7 North York General Hospital, Toronto, Ontario, Canada; 8 Toronto Western Family Health Team, University Health Network, Toronto, Ontario, Canada; 9 Women’s College Research Institute, Women’s College Hospital, Toronto, Ontario, Canada; Bay Area Hospital, North Bend Medical Center, UNITED STATES

## Abstract

**Background:**

The global burden of Parkinson’s disease (PD) has more than doubled over the past three decades, and this trend is expected to continue. Despite generally poorer access to health care services in rural areas, little previous work has examined health system use in persons with PD by rurality. We examined trends in the prevalence of PD and health service use among persons with PD by rurality in Ontario, Canada.

**Methods:**

We conducted a repeated, cross-sectional analysis of persons with prevalent PD aged 40+ years on April 1^st^ of each year from 2000 to 2018 using health administrative databases and calculated the age-sex standardized prevalence of PD. Prevalence of PD was also stratified by rurality and sex. Negative binomial models were used to calculate rate ratios with 95% confidence intervals comparing rates of health service use in rural compared to urban residents in 2018.

**Results:**

The age-sex standardized prevalence of PD in Ontario increased by 0.34% per year (p<0.0001) and was 459 per 100,000 in 2018 (n = 33,479), with a lower prevalence in rural compared to urban residents (401 vs. 467 per 100,000). Rates of hospitalizations and family physician visits declined over time in both men and women with PD in rural and urban areas, while rates of emergency department, neurologist, and other specialist visits increased. Adjusted rates of hospitalizations were similar between rural and urban residents (RR = 1.04, 95% CI [0.96, 1.12]), while rates of emergency department visits were higher among rural residents (RR = 1.35, 95% CI [1.27, 1.42]). Rural residents had lower rates of family physician (adjusted RR = 0.82, (95% CI [0.79, 0.84]) and neurologist visits (RR = 0.74, 95% CI [0.72, 0.77]).

**Interpretation:**

Lower rates of outpatient health service use among persons residing in rural regions, contrasting with higher rates of emergency department visits suggest inequities in access. Efforts to improve access to primary and specialist care for persons with PD in rural regions are needed.

## Introduction

The worldwide burden of Parkinson’s disease (PD) has more than doubled over the past three decades with an estimated 6 in 1,000 persons in North America living with the condition, making it an important public health concern [1, 2]. PD is a neurodegenerative disorder resulting in a broad spectrum of motor and non-motor symptoms whose incidence rises with age [3]. Previous studies have shown that persons with PD have higher health system use than those without after adjustment for age and sex [4–6], contributing to high costs for health care systems [7, 8]. The determinants of health service use for people with PD are not well understood and relatively few studies have examined how health service use among persons with PD differs with respect to rural and urban residence and socioeconomic status [9, 10]. One study found that higher socioeconomic status was associated with higher rates of hospitalizations among men with PD [9]. The incidence and prevalence of PD in urban areas was found to be higher in the lowest income quintile neighbourhoods in Manitoba, Canada [10]. While the prevalence of PD was also higher in rural areas with the lowest income quintile, no association was noted for incidence [10].

In the general population, living in rural areas has been associated with poorer access to important health services, worse health outcomes, and lower life expectancy [11–13]. Persons residing in rural regions may also experience lower incomes and lower rates of educational attainment that place them at an increased risk of poor health outcomes [11]. A better understanding of the differences in health service use among persons with PD by rurality would enable appropriate health policy and health service planning to meet the needs of these populations.

Therefore, the objectives of our study were to compare trends in the prevalence of PD in Ontario, Canada over time and to examine health service use among persons with PD by rural versus urban residence.

## Methods

### Study design, setting and data sources

We conducted a repeated, cross-sectional analysis of the prevalence of PD and associated health service use using linked health administrative databases in Ontario, Canada. These datasets were linked using encoded identifiers and analyzed at ICES. In 2018, the institute formerly known as the Institute for Clinical Evaluative Sciences formally adopted the initialism ICES as its official name. ICES is an independent, nonprofit research institute whose legal status under Ontario’s health information privacy law allows it to collect and analyze health care and demographic data, without consent, for health system evaluation and improvement. Ontario is Canada’s most populous province, with a population of approximately 14.9 million persons. Ontario residents have universal, publicly funded access to necessary physician services, hospital care, and essential home-based health care services under the province’s health insurance plan (see **Table 1** in **S1 Appendix** for full list of databases).

### Study population

We identified all persons in Ontario aged 40 years and older who were alive and living with PD on April 1^st^ of each year from 2000 to 2018 using a previously validated health administrative data algorithm. PD was defined as: three physician billing codes for PD (diagnosis code: 332) in two years each separated by at least 30 days [14] using physician billing data until April 1^st^ 2020. This algorithm was found to have good performance characteristics when validated against a review of family physician electronic medical record charts as the reference standard (sensitivity: 72.3%, specificity: 100%, positive predictive value: 82.1%, negative predictive value: 99.9% (**Table 2 in S1 Appendix**)) and is a refinement of a previous, broader algorithm for Parkinsonism [15].

On April 1^st^ of each year we identified age, sex, neighbourhood income quintile, 18 co-existing chronic conditions (identified using validated health administrative data algorithms [16]) and whether or not the individual resided in a long-term care facility. Neighbourhood income quintile was defined by linking to Statistics Canada census data using postal codes and is based on the ranking of median income by region [17].

### Rurality

The main exposure was rural versus urban location of residence. Rural was defined as living in a rural or small town community with a population size of <10,000 persons outside the commuting zone of larger urban centres according to definitions from Statistics Canada [18].

### Health service use

For all persons with PD on April 1^st^ of each year we calculated rates of health services use from April 1^st^ to March 31^st^ for hospitalizations, emergency department visits, family physician visits, neurologist visits, other specialist visits (excluding neurologists), rehabilitation admissions, home care visits and long-term care admissions. Persons residing in long-term care on April 1^st^ were excluded from the denominator for home care visit and long-term care admission rates as persons in long-term care generally do not receive home care services and are no longer at risk of admission to long-term care, respectively.

### Statistical analysis

Standardized differences were used to compare characteristics of persons with prevalent PD by rurality within 2000 and 2018 study years and to compare persons with PD in rural regions across 2000 and 2018 study years [19]. A standardized difference (s-diff) >0.10 was considered a meaningful imbalance. We calculated the overall age-sex standardized prevalence of PD per 100,000 persons aged 40 years and older for each year, as well as age-standardized prevalence by rurality-sex strata to account for known differences in PD incidence, disease progression and outcomes by sex [20]. The standard population was the 2016 Ontario census population [21]. We measured age-standardized rates of health service use per 100 person-years for each year stratified by rurality-sex. For health service use outcomes, individuals were censored on death or loss of health insurance eligibility. The average annual percent change in rates of each health service were calculated using a Poisson model including the count of health service use from the age-standardized rate as the dependent variable and year as the independent variable with the log of the person-years in each year included as the offset term. P-values were used to assess the significance of the annual percent change and were 2-tailed, with *p* < 0.05 considered statistically significant. Multivariable negative binomial and log binomial models were used to estimate rate and risk ratios and 95% confidence intervals for health service use count and binary outcomes (rehabilitation and long-term care admission only), respectively, during the latest study year (April 1^st^ 2018 to March 31^st^ 2019) comparing rural to urban residents.

We estimated three sets of models; unadjusted, age-/sex-adjusted, and fully-adjusted models including income quintile and individual comorbidities. All analyses were conducted using SAS Enterprise Guide Version 7.1 (SAS Institute Inc.).

### Ethics statement

The use of data in this project was authorized under section 45 of Ontario’s Personal Health Information Protection Act (PHIPA) and does not require review by a Research Ethics Board.

## Results

### Prevalence of Parkinson’s disease

In 2018, the age-sex standardized prevalence of PD was 459 per 100,000 persons. We found a 14% lower prevalence of PD in persons residing in rural compared to urban areas (401 vs. 467 per 100,000).

**Fig 1:**
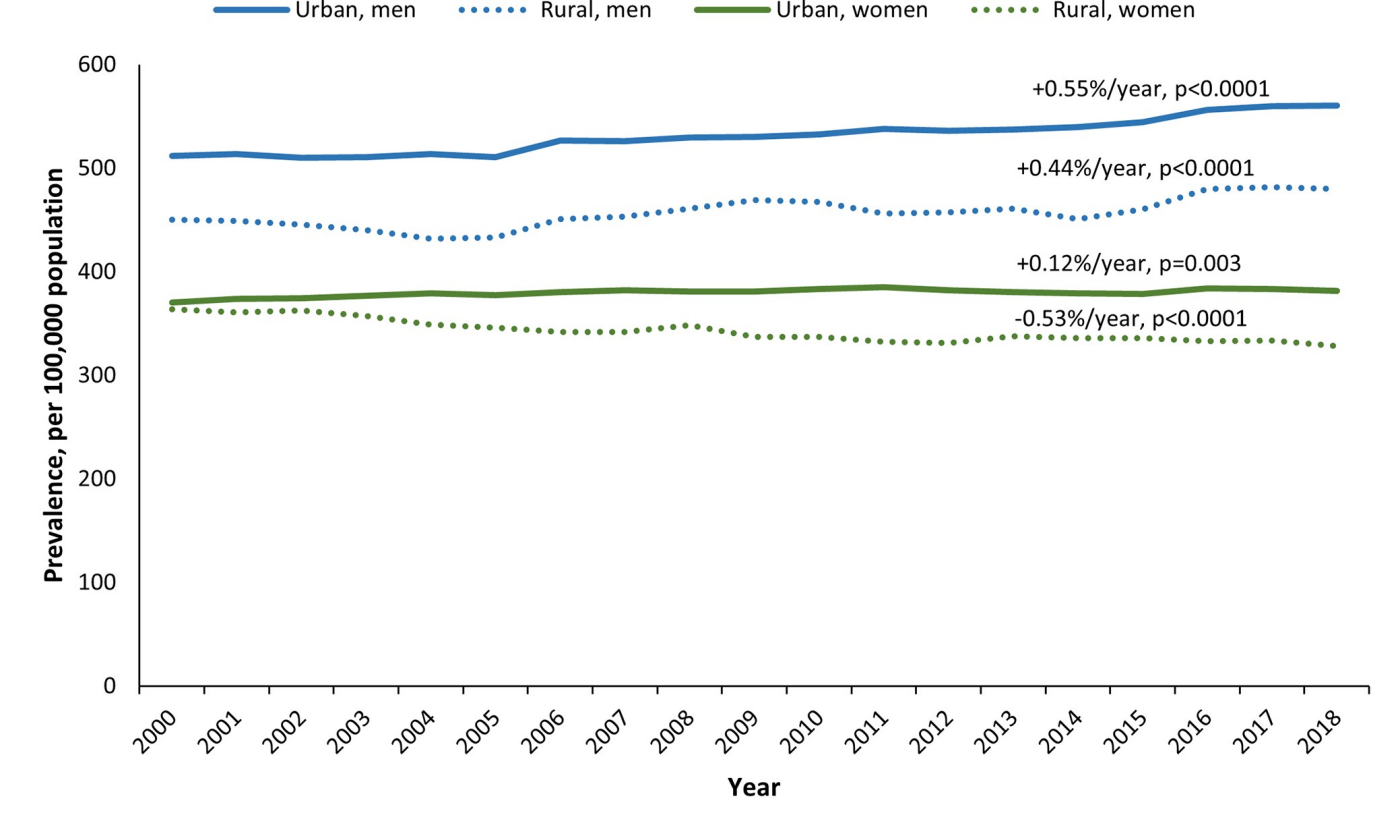
Age-standardized prevalence of Parkinson’s disease among persons aged 40 and older in Ontario, Canada from 2000 to 2018, by rurality and sex*. *Age-standardized prevalence to the 2018 Ontario population.

The age-standardized prevalence was 47% higher among men than among women (550 vs. 375 per 100,000). Within both men and women, we also found a lower prevalence of PD in rural compared to urban areas (men: 480 vs. 561 per 100,000; women: 328 vs. 381 per 100,000). From 2000 to 2018, we observed a modest increase in the prevalence of PD within urban and rural regions by sex, with the exception of women residing in rural regions for which we observed a modest decline (**Fig 1**).

### Characteristics of persons with Parkinson’s disease

We identified a total of 33,479 persons with prevalent PD as of April 1^st^ 2018 and 3,578 (10.7%) resided in rural areas **(Table 1).** In 2018, persons with PD residing in rural and urban areas were of similar age (median 74 years vs. 75 years, s-diff = 0.07) and were similarly likely to reside in a long-term care home (16.6% vs. 15.9%, s-diff = 0.02). Persons with PD living in rural and urban areas were similarly likely to live in the lowest (22.1% vs. 20.9%, s-diff = 0.03) and highest income quintiles (17.1% vs. 20.4%, s-diff = 0.08). Rural residents with PD were more likely to have comorbid chronic obstructive pulmonary disease (23.6% vs. 19.6%, s-diff = 0.10), but had a modestly lower number of chronic conditions (mean = 2.3 vs. 2.5, s-diff = 0.18). PD duration was similar between rural and urban residents (mean 6.4 years vs. 6.5 years, s-diff = 0.02).

**Table 1:** Characteristics of persons with prevalent Parkinson’s disease (PD) in Ontario, Canada by year and rurality.

	Persons with prevalent PD—2000			Persons with prevalent PD—2018			
Characteristic*	Urban	Rural	St diff^†^	Urban	Rural	St diff^†^	St diff^‡^
	(N = 15,943)	(N = 2,567)	Urban vs. Rural	(N = 29,901)	(N = 3,578)	Urban vs. Rural	Rural 2000 vs. 2018
**Age**							
Mean (±SD)	74.3 ± 10.0	74.5 ± 10.2	0.03	74.6 ± 10.5	73.9 ± 10.1	0.07	0.06
Median (IQR)	76.0 (69.0–81.0)	76.0 (69.0–81.0)	0.03	75.0 (68.0–82.0)	74.0 (67.0–81.0)	0.08	0.08
40–54 years	778 (4.9%)	118 (4.6%)	0.01	1,261 (4.2%)	134 (3.7%)	0.02	0.04
55–64 years	1,708 (10.7%)	279 (10.9%)	0.01	3,759 (12.6%)	502 (14.0%)	0.04	0.10
65–74 years	4,704 (29.5%)	749 (29.4%)	0.01	8,867 (29.7%)	1,155 (32.3%)	0.06	0.07
75–84 years	6,514 (40.9%)	1,025 (39.9%)	0.02	10,586 (35.4%)	1,244 (34.8%)	0.01	0.11
≥85 years	2,239 (14.0%)	396 (15.4%)	0.04	5,428 (18.2%)	543 (15.2%)	0.08	0.007
**Sex, n (%)**							
Female	7,531 (47.2%)	1,213 (47.3%)	0.0003	12,843 (43.0%)	1,426 (39.9%)	0.06	0.15
Male	8,412 (52.8%)	1,354 (52.7%)	0.0003	17,058 (57.0%)	2,152 (60.1%)	0.06	0.15
**Income quintile**							
1 (Lowest)	3,212 (20.1%)	556 (22.0%)	0.05	6,259 (20.9%)	790 (22.1%)	0.03	0.002
2	3,507 (22.0%)	557 (21.7%)	0.007	6,107 (20.4%)	771 (21.5%)	0.03	0.004
3	3,127 (19.6%)	489 (19.0%)	0.01	5,857 (19.6%)	722 (20.2%)	0.01	0.03
4	2,803 (17.6%)	520 (20.3%)	0.07	5,593 (18.7%)	682 (19.1%)	0.009	0.03
5 (Highest)	3,294 (20.7%)	436 (17.0%)	0.09	6,085 (20.4%)	613 (17.1%)	0.08	0.004
**Long-term care**	3,172 (19.9%)	592 (23.1%)	0.08	4,748 (15.9%)	593 (16.6%)	0.02	0.16
**Duration of PD (years)**							
Mean (±SD)	4.49 ± 2.91	4.52 ± 2.88	0.01	6.51 ± 5.66	6.39 ± 5.79	0.02	0.41
Median (IQR)	4.20 (1.81–7.45)	4.14 (1.89–7.43)	0.02	4.89 (2.16–9.38)	4.52 (1.98–9.38)	0.04	0.20
**Chronic conditions**							
Diabetes	2,548 (16.0%)	369 (14.4%)	0.04	8,958 (30.0%)	968 (27.1%)	0.06	0.32
Hypertension	7,840 (49.2%)	1,314 (51.2%)	0.04	20,310 (67.9%)	2,291 (64.0%)	0.08	0.26
Asthma	1,300 (8.2%)	199 (7.8%)	0.01	4,370 (14.6%)	468 (13.1%)	0.04	0.18
COPD	2,728 (17.1%)	522 (20.3%)	0.08	5,854 (19.6%)	846 (23.6%)	0.10	0.08
CHF	2,072 (13.0%)	384 (15.0%)	0.06	3,309 (11.1%)	424 (11.9%)	0.02	0.09
Previous AMI	803 (5.0%)	148 (5.8%)	0.03	1,971 (6.6%)	262 (7.3%)	0.03	0.06
Previous stroke/TIA	1,063 (6.7%)	204 (7.9%)	0.05	1,672 (5.6%)	220 (6.1%)	0.02	0.07
Peripheral vascular disease	427 (2.7%)	66 (2.6%)	0.007	564 (1.9%)	71 (2.0%)	0.007	0.04
Chronic kidney disease	604 (3.8%)	70 (2.7%)	0.06	3,623 (12.1%)	357 (10.0%)	0.07	0.30
Rheumatoid arthritis	218 (1.4%)	40 (1.6%)	0.02	705 (2.4%)	88 (2.5%)	0.007	0.06
Dementia	3,015 (18.9%)	458 (17.8%)	0.03	8,593 (28.7%)	872 (24.4%)	0.099	0.16
**Number of chronic conditions**							
Mean (±SD)	2.29 ± 1.71	2.19 ± 1.61	0.06	2.53 ± 1.66	2.25 ± 1.58	0.18	0.03
Median (IQR)	2.00 (1.00–3.00)	2.00 (1.00–3.00)	0.04	2.00 (1.00–4.00)	2.00 (1.00–3.00)	0.18	0.04
0–1 conditions	5,938 (37.2%)	988 (38.5%)	0.03	8,709 (29.1%)	1,275 (35.6%)	0.14	0.06
2 conditions	3,744 (23.5%)	597 (23.3%)	0.005	7,434 (24.9%)	927 (25.9%)	0.02	0.06
3 conditions	2,851 (17.9%)	497 (19.4%)	0.04	6,259 (20.9%)	652 (18.2%)	0.07	0.03
4 conditions	1,672 (10.5%)	252 (9.8%)	0.02	3,858 (12.9%)	406 (11.3%)	0.05	0.05
5+ conditions	1,738 (10.9%)	233 (9.1%)	0.06	3,641 (12.2%)	318 (8.9%)	0.11	0.007

St diff = Standardized difference; SD = Standard Deviation; IQR = Interquartile Range; COPD = Chronic Obstructive Pulmonary Disease; CHF = Congestive Heart Failure; AMI = Acute Myocardial Infarction; TIA = Transient Ischemic Attack.

*Data are presented as frequency (%) unless otherwise indicated

Standardized difference scores measure the effect size between two groups, independent of sample size: ^†^ compares urban and rural persons with Parkinson’s disease within 2000 and 2018, respectively and ^‡^ compares rural persons with Parkinson’s disease in 2000 to those in 2018.

When comparing persons with prevalent PD residing in rural areas in 2000 compared to 2018, those in the more recent time period were more likely to be male (60.1% vs. 52.7%, s-diff = 0.15), to have dementia (24.4% vs. 17.8%, s-diff = 0.16), and had longer duration of PD (median: 4.52 vs. 4.14 years, s-diff = 0.20), but were less likely to reside in long-term care (16.6% vs. 23.1%, s-diff = 0.16).

### Trends in health service use over time

**Fig 2:**
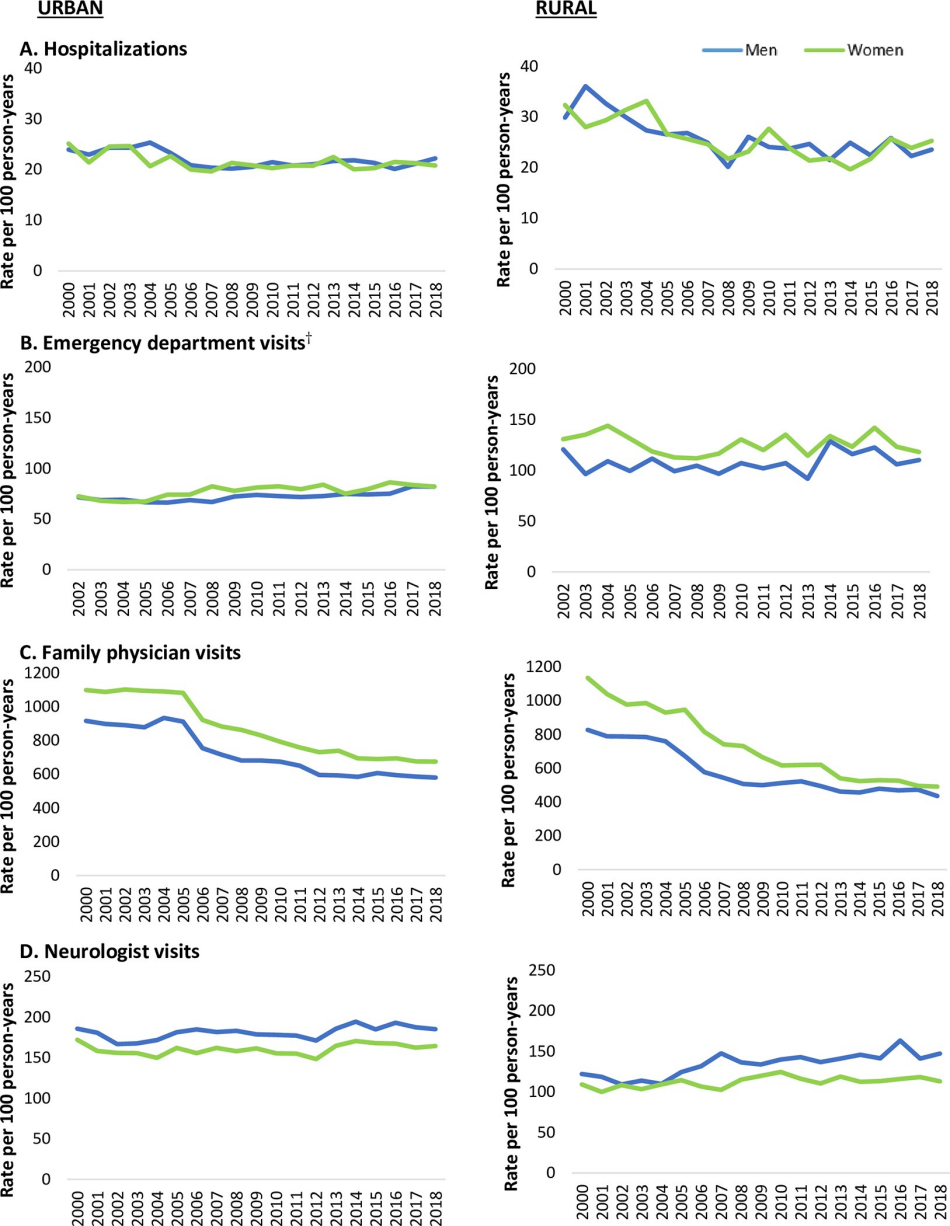
Age-standardized rates of health service use among persons aged 40 and older with prevalent Parkinson’s disease in Ontario, Canada from 2000 to 2018, by rurality and sex*. *Age-standardized rate measured per 100 person-years, standardized to the 2018 Ontario population. ^†^Emergency department visit data available starting in 2002.

Rates of hospitalizations declined over time (**Fig 2**) among both rural and urban residents (men-rural = -1.87%, p<0.001 men urban = -0.68%, p<0.001, women rural = -1.83%, p<0.001, women urban = -0.62%, p<0.001). Emergency department visit, neurologist visit, and other specialist visit rates showed modest increases over time across most sex-rurality strata. Rates of family physician visits declined more rapidly over time in men and women residing in rural areas compared to urban areas (men-rural = -3.68%, p<0.001 men urban = - 3.00%, p<0.001, women rural = -4.87%, p<0.001, women urban = -3.29%, p<0.001). Rates of rehabilitation admissions were stable over time. Home care visits showed increases over time for all sex-rurality groups, while rates of long-term care admission declined significantly over time, with the exception of men living in rural areas, for whom rates were relatively stable (men-rural, = -0.92%, p = 0.12, men-urban, = -2.02%, p<0.0001, women-rural = -1.90%, p = 0.002, women urban = -1.64%, p<0.0001).

### Intensity of health service use by rurality

After adjustment for relevant confounders, persons with PD residing in rural areas had similar rates of hospitalizations (adjusted RR = 1.04, 95% CI [0.96, 1.12]), but a 35% increased rate of emergency department visits (adjusted RR = 1.35, 95% CI [1.27, 1.42]) compared to persons with PD living in urban areas (**Table 2**). Rural residents had an 18% lower rate of family physician visits (adjusted RR = 0.82, (95% CI [0.79, 0.84]), a 26% lower rate of neurologist visits (adjusted RR = 0.74, 95% CI [0.72, 0.77]), and a 31% lower rate of other specialist visits (adjusted RR = 0.69, 95% CI [0.66, 0.72]) compared to urban residents. Rates of rehabilitation admission were 39% less frequent (adjusted RR = 0.61, 95% CI [0.46,0.81]), while rates of home care visits were 34% lower among persons residing in rural compared to urban areas (adjusted RR = 0.66, 95% CI [0.58, 0.76]). Rural residents had a 28% increased rate of long-term care admission (adjusted RR = 1.28, 95% CI [1.08, 1.50]).

**Table 2:** Rate ratios of health service use comparing rural to urban persons with prevalent Parkinson’s disease as of April 1, 2018.

	Rate Ratio (95% CI)		
Health service use (estimate for rural; reference: urban)	Unadjusted	Adjusted for age group, sex	Fully Adjusted*
**Hospitalizations**	1.00 (0.92–1.08)	1.02 (0.94–1.11)	1.04 (0.96–1.12)
**Emergency department visits**	1.31 (1.23–1.38)	1.32 (1.25–1.40)	1.35 (1.27–1.42)
**Family physician visits**	0.80 (0.77–0.83)	0.80 (0.78–0.83)	0.82 (0.79–0.84)
**Neurologist visits**	0.77 (0.74–0.80)	0.74 (0.71–0.77)	0.74 (0.72–0.77)
**Other specialist visits**	0.70 (0.67–0.73)	0.69 (0.66–0.73)	0.69 (0.66–0.72)
**Rehabilitation admissions**	0.60 (0.46–0.80)	0.61 (0.46–0.81)	0.61 (0.46–0.81)
**Home care visits**	0.60 (0.52–0.69)	0.64 (0.56–0.73)	0.66 (0.58–0.76)
**Long-term care admissions**	1.10 (0.94–1.29)	1.17 (1.00–1.37)	1.28 (1.08–1.50)

CI = Confidence Interval; ref = Reference Group

*Adjusted for age group, sex, income quintile, index year, diabetes, hypertension, asthma, chronic obstructive pulmonary disease, congestive heart failure, previous acute myocardial infarction, previous stroke/transient ischemic attack, peripheral vascular disease, chronic kidney disease, rheumatoid arthritis, and dementia.

## Discussion

In the present study, we found a lower prevalence of PD in rural residents compared to urban residents, with generally increasing trends in prevalence over time (with the exception of women residing in rural regions). Among persons with PD, we observed declines in the rates of hospitalizations, family physician visits, and long-term care admission over time within both rural and urban regions by sex, while rates of other health services (i.e. emergency department, neurologist, and other specialist visits, rehabilitation admissions) remained stable or increased (i.e., home care visits). After adjustment for age, sex, income quintile, year and comorbidities, rates of rates of outpatient health care use (i.e., family physician, neurologist and other specialist visits) and rehabilitation admissions were significantly lower among persons in rural areas, while rates of emergency department visits and long-term care admissions were higher in persons with PD residing in rural areas.

The prevalence of PD among persons aged 40 years and older we observed was similar to estimates from other jurisdictions [22, 23]. Although the evidence is mixed, some previous studies have found a higher prevalence of PD in urban areas [23]; including studies from the UK [24–26], Taiwan [23, 27, 28], and China [29]. A UK study showed a higher incidence of PD diagnosed in primary care settings in urban compared to rural regions [26]. A Chinese cross-sectional survey of urban and rural counties showed that the prevalence of PD among persons aged 65 years and older was higher in urban compared to rural counties (1.98% vs. 1.48%) [29]. However, recent Australian and Latin American studies have found no difference in the prevalence of PD in rural and urban regions, after adjustment for demographic characteristics [30, 31]. Although the exact cause of PD is unknown, genetic and environment factors likely both play a role, including exposures associated with agricultural and industrial occupations [32]. It is possible that higher prevalence of PD observed in urban residents in our study may be related to exposures associated with urban living and/or differences in lifestyle and health system factors. Persons residing in urban areas may have had an increased likelihood of detection of PD due to easier access to specialist care and other healthcare services, which tend to be concentrated in urban areas in Canada [33]. Our finding of a modest increase in PD prevalence over time is congruent with recent studies that have found increasing PD prevalence [1].

Declines in health service utilization, particularly in rates of hospitalization and family physicians over the study period mirror trends seen in other chronic disease populations in Ontario, including hospitalizations for complications of diabetes [34], cardiovascular disease hospitalizations [35], and asthma [36], suggesting that health system factors are playing a role in the observed trends. Declining rates of family physician visits can be attributed to the shift to patient enrollment models in Ontario starting in 2001, whereby changes were made to how physicians were compensated (generally changing from fee-for service per visit compensation to blended capitation models where physicians are paid per patient enrolled in their practice, adjusted for age and sex) [37]. This shift in physician reimbursement has been shown to be associated with fewer visits to family physicians, but no differences in quality of care for persons with chronic diseases [38]. It is possible that improvements in treatments for PD and other conditions are contributing to lower rates of hospitalizations observed.

Relatively few studies have examined differences in rates of health care use among persons with PD by rurality. Similar to our findings, a study examining health care use among Manitoba residents with PD found that rural residence was associated with a lower rate of family physician and specialist visits, but found higher rates of hospitalizations [4], while no differences in hospitalization rates were observed in the present study. An analysis of US Medicare beneficiaries with PD also found that rural residence was associated with a decreased frequency of neurologist visits [39].

In Canada very few neurologists and other specialists practice in rural areas [40], which may contribute to the lower rates observed in this study. Persons with PD residing in rural areas in Canada and the United States have expressed difficulties accessing specialist care—due to lack of specialists in their region and having to travel long distances to receive care [41, 42]. The large geography of Ontario and centralization of neurologist care in academic centres in southern, urban areas poses difficulties for rural and northern patients trying to access specialist care. Telehealth has been proposed as a potential solution to improve access to specialist care by persons with PD residing in rural regions, although there are concerns regarding the ability of neurologists to assess patients remotely [41]. The Ontario Telemedicine Network (OTN) provides infrastructure to allow for remote neurologist consultations at thousands of locations across Ontario, however, only 18% of total calls to rural areas were for internal medicine based on data from 2008–2014 [43]. During the COVID-19 pandemic, which post-dates our study, there was a rapid shift to virtual and telephone physician care for family physicians, neurologists, and other specialists associated with the introduction of new provincial billing codes [44]. Virtual care has the potential to reduce barriers for persons residing in rural areas to improve access to physician services.

While increased supply and continuity of primary care has been shown to be associated with improved health outcomes at the population and individual levels [45], to our knowledge, there is limited evidence regarding the association in persons with PD. This is an important area for further study. Increased access to neurologist care has been shown to improve patient outcomes and reduce potentially unnecessary health system use and costs for persons with PD [39, 46, 47]. More frequent neurologist care has been associated with decreases in hospitalizations, emergency department visits, and days spent in skilled nursing facilities [39, 46]. Specifically, neurologist care was associated with a decreased risk of hospitalization due to complications of PD, including psychosis, urinary tract infection, and traumatic injury as well as recurrent hospitalizations [39]. In previous research, health care expenditures for inpatient care, home care, skilled nursing facility care, hospice services, and medical equipment were lower for patients with higher levels of neurologist involvement [46]. Neurologist care has also been associated with lower rates of nursing home placement, hip fractures and mortality [47]. Increased access to neurologist care for rural residents with PD in Ontario may reduce the use of more costly health care services.

Lower rates of rehabilitation use in rural residents with PD may reflect increased barriers experienced by rural residents accessing this care such as distance, cost, and transportation issues. Similar issues have been noted by patients attending cardiac rehabilitation in Ontario [48]. Similarly, lower rates of home care visits in rural regions are likely due to challenges related to low population density and large service delivery areas [49]. In the present study, we noted increased rates of long-term care admission in persons with PD in rural regions, which may be related to poorer access to home care services and other health care services for persons needed to support individuals to live longer in community settings [50].

### Limitations and other considerations

Although our study had several strengths, there are some limitations that are worthy of consideration. Though we used a validated health administrative algorithm to identify persons with PD, it is likely that we may have under-captured persons with PD, and would not capture those who may have not yet sought care or been diagnosed. It is also possible that the algorithm identified some persons with the broader condition of parkinsonism (i.e., a group of neurological disorders that cause movement difficulties) and it is unclear whether this is differential between rural and urban residents. In addition, there may be residual differences in the severity of PD (which we are unable to measure) or other characteristics between persons with PD residing in rural and urban areas that may have influenced health service use. Finally, we did not examine health care use specifically for PD care, and thus the health care use trends represent the combined effects of PD and its comorbidities.

## Conclusions

In this population-based study of persons with PD, we found a lower prevalence of PD in rural areas, as well as lower rates of family physician, neurologist, other specialist and home care visits, but higher rates of emergency department visits and long-term care admission. These findings suggest inequities in health system access for persons with PD living in rural regions. Given the associations between neurologist care and better outcomes in people with PD, future work should focus on understanding and overcoming barriers to specialist care, particularly in rural areas. It is also likely that reduced access to primary care in rural areas is having a negative impact on the health of people with PD, although this remains to be investigated.

## Supporting information

S1 Appendix Trends in health service use among persons with Parkinson’s disease by rurality: A population-based repeated cross-sectional study. (DOCX)

## References

[1] Global Burden of Disease 2016 Parkinson’s Disease Collaborators. Global, regional and national burden of Parkinson’s disease, 1990–2016: a systematic analysis for the Global Burden of Disease Study 2016. The Lancet Neurology. 2016;17(11):939–53. 10.1016/S1474-4422(18)30295-3 PMC6191528 30287051

[2] Marras C, Beck J, Bower J, Roberts E, Ritz B, Ross G, et al. Prevalence of Parkinson’s disease across North America. NPJ Parkinson’s disease. 2018;4(1):1–7. doi: 10.1038/s41531-018-0058-0 30003140 PMC6039505

[3] Van Den Eeden SK, Tanner CM, Bernstein AL, Fross RD, Leimpeter A, Bloch DA, et al. Incidence of Parkinson’s Disease: Variation by Age, Gender, and Race/Ethnicity. American Journal of Epidemiology. 2003;157(11):1015–22. doi: 10.1093/aje/kwg068 12777365

[4] Hobson DE, Lix LM, Azimaee M, Leslie WD, Burchill C, Hobson S. Healthcare utilization in patients with Parkinson’s disease: a population-based analysis. Parkinsonism Relat Disord. 2012;18(8):930–5. doi: 10.1016/j.parkreldis.2012.04.026 22621819

[5] Noyes K, Liu H, Li Y, Holloway R, Dick AW. Economic burden associated with Parkinson’s disease on elderly Medicare beneficiaries. 2006;21(3):362–72. 10.1002/mds.20727 16211621

[6] Heinzel S, Berg D, Binder S, Ebersbach G, Hickstein L, Herbst H, et al. Do We Need to Rethink the Epidemiology and Healthcare Utilization of Parkinson’s Disease in Germany? Frontiers in neurology. 2018;9:500. doi: 10.3389/fneur.2018.00500 30008693 PMC6033992

[7] von Campenhausen S, Winter Y, e Silva AR, Sampaio C, Ruzicka E, Barone P, et al. Costs of illness and care in Parkinson’s disease: an evaluation in six countries. European Neuropsychopharmacology. 2011;21(2):180–91. doi: 10.1016/j.euroneuro.2010.08.002 20888737

[8] Hagell P, Nordling S, Reimer J, Grabowski M, Persson U. Resource use and costs in a Swedish cohort of patients with Parkinson’s disease. Movement disorders: official journal of the Movement Disorder Society. 2002;17(6):1213–20. doi: 10.1002/mds.10262 12465059

[9] Li X, Sundquist J, Sundquist K. Socioeconomic and occupational groups and Parkinson’s disease: a nationwide study based on hospitalizations in Sweden. International archives of occupational and environmental health. 2009;82(2):235–41. doi: 10.1007/s00420-008-0327-z 18427829

[10] Lix LM, Hobson DE, Azimaee M, Leslie WD, Burchill C, Hobson S. Socioeconomic variations in the prevalence and incidence of Parkinson’s disease: a population-based analysis. J Epidemiol Community Health. 2010;64(4):335–40. doi: 10.1136/jech.2008.084954 19679711

[11] (PHAC) PHAoC, Research CfRaNH, (CIHI) CIfHI. How Healthy Are Rural Canadians? An Assessment of Their Health Status and Health Determinants. Toronto, ON; 2006.

[12] Income and Health: Opportunities to achieve health equity in Ontario. Toronto, ON; 2016.

[13] McIntosh CN, Finès P, Wilkins R, Wolfson MC. Income disparities in health-adjusted life expectancy for Canadian adults, 1991 to 2001December 2009; Catalogue no. 82-003-XPE • Health Reports, Vol. 20, no. 4. 20108606

[14] Butt D, Tu K. Validated health administrative algorithm for Parkinson’s disease. 2020.

[15] Butt DA, Tu K, Young J, Green D, Wang M, Ivers N, et al. A validation study of administrative data algorithms to identify patients with Parkinsonism with prevalence and incidence trends. Neuroepidemiology. 2014;43(1):28–37. doi: 10.1159/000365590 25323155

[16] Mondor L, Maxwell CJ, Hogan DB, Bronskill SE, Gruneir A, Lane NE, et al. Multimorbidity and healthcare utilization among home care clients with dementia in Ontario, Canada: a retrospective analysis of a population-based cohort. PLoS medicine. 2017;14(3):e1002249. doi: 10.1371/journal.pmed.1002249 28267802 PMC5340355

[17] Statistics Canada. Postal CodeOM Conversion File (PCCF), Reference Guide, 2017; Catalogue no. 92-154-G.

[18] du Plessis V, Beshiri R, Bollman RD, Clemenson H. Definitions of Rural Ottawa, Ontario: Statistics Canada; 2001 [Available from: https://www150.statcan.gc.ca/n1/en/pub/21-006-x/21-006-x2001003-eng.pdf?st=pP7Z0CBd.

[19] Austin PC. The relative ability of different propensity score methods to balance measured covariates between treated and untreated subjects in observational studies. Medical Decision Making. 2009;29(6):661–77. doi: 10.1177/0272989X09341755 19684288

[20] Cerri S, Mus L, Blandini F. Parkinson’s disease in women and men: What’s the difference? Journal of Parkinson’s disease. 2019;9(3):501–15. 10.3233/JPD-191683 PMC6700650 31282427

[21] Ontario Ministry of Health and Long-Term Care IntelliHEALTH ONTARIO. Population Estimates. 2018 Date Data Last Refreshed [April/2022].

[22] Pringsheim T, Jette N, Frolkis A, Steeves TD. The prevalence of Parkinson’s disease: a systematic review and meta-analysis. Mov Disord. 2014;29(13):1583–90. doi: 10.1002/mds.25945 24976103

[23] Chen CC, Chen TF, Hwang YC, Wen YR, Chiu YH, Wu CY, et al. Different prevalence rates of Parkinson’s disease in urban and rural areas: a population-based study in Taiwan. Neuroepidemiology. 2009;33(4):350–7. doi: 10.1159/000254572 19887842

[24] Hobson P, Gallacher J, Meara J. Cross‐sectional survey of Parkinson’s disease and parkinsonism in a rural area of the United Kingdom. Movement disorders: official journal of the Movement Disorder Society. 2005;20(8):995–8. doi: 10.1002/mds.20489 15852368

[25] Schrag A, Ben-Shlomo Y, Quinn N. Cross sectional prevalence survey of idiopathic Parkinson’s disease and Parkinsonism in London. Bmj. 2000;321(7252):21–2. doi: 10.1136/bmj.321.7252.21 10875828 PMC27420

[26] Horsfall L, Petersen I, Walters K, Schrag A. Time trends in incidence of Parkinson’s disease diagnosis in UK primary care. Journal of neurology. 2013;260(5):1351–7. doi: 10.1007/s00415-012-6804-z 23263597

[27] Chen R, Chang S, Su C, Chen T, Yen M, Wu H, et al. Prevalence, incidence, and mortality of PD: a door-to-door survey in Ilan county, Taiwan. Neurology. 2001;57(9):1679–86. 11706111 10.1212/wnl.57.9.1679

[28] Wang S-J, Fuh J-L, Teng EL, Liu C-Y, Lin K-P, Chen H-M, et al. A door-to-door survey of Parkinson’s disease in a Chinese population in Kinmen. Archives of neurology. 1996;53(1):66–71. doi: 10.1001/archneur.1996.00550010084020 8599561

[29] Song Z, Liu S, Li X, Zhang M, Wang X, Shi Z, et al. Prevalence of Parkinson’s Disease in Adults Aged 65 Years and Older in China: A Multicenter Population-Based Survey. Neuroepidemiology. 2022;56(1):50–8. doi: 10.1159/000520726 34758470

[30] Ayton D, Ayton S, Barker AL, Bush AI, Warren N. Parkinson’s disease prevalence and the association with rurality and agricultural determinants. Parkinsonism & related disorders. 2019;61:198–202. doi: 10.1016/j.parkreldis.2018.10.026 30377035

[31] Llibre-Guerra JJ, Prina M, Sosa AL, Acosta D, Jimenez-Velazquez IZ, Guerra M, et al. Prevalence of parkinsonism and Parkinson disease in urban and rural populations from Latin America: A community based study. The Lancet Regional Health-Americas. 2022;7:100136. doi: 10.1016/j.lana.2021.100136 35300390 PMC8920908

[32] Kieburtz K, Wunderle KB. Parkinson’s disease: evidence for environmental risk factors. Movement Disorders. 2013;28(1):8–13. doi: 10.1002/mds.25150 23097348

[33] Information CIfH. Geographic Distribution of Physicians in Canada: Beyond How Many and Where. Ottawa, Ontario; 2005.

[34] Booth GL, Hux JE, Fang J, Chan BT. Time trends and geographic disparities in acute complications of diabetes in Ontario, Canada. Diabetes care. 2005;28(5):1045–50. 15855565 10.2337/diacare.28.5.1045

[35] Tu JV, Khan AM, Ng K, Chu A. Recent temporal changes in atherosclerotic cardiovascular diseases in Ontario: clinical and health systems impact. Canadian Journal of Cardiology. 2017;33(3):378–84. doi: 10.1016/j.cjca.2016.11.009 28129964

[36] Crighton EJ, Mamdani MM, Upshur RE. A population based time series analysis of asthma hospitalisations in Ontario, Canada: 1988 to 2000. BMC Health Services Research. 2001;1(1):7. doi: 10.1186/1472-6963-1-7 11580873 PMC57008

[37] Glazier RH, Klein-Geltink J, Kopp A, Sibley LM. Capitation and enhanced fee-for-service models for primary care reform: a population-based evaluation. Cmaj. 2009;180(11):E72–E81. doi: 10.1503/cmaj.081316 19468106 PMC2683211

[38] Tummalapalli SL, Estrella MM, Jannat-Khah DP, Keyhani S, Ibrahim S. Capitated versus fee-for-service reimbursement and quality of care for chronic disease: a US cross-sectional analysis. BMC health services research. 2022;22(1):1–12. 34980111 10.1186/s12913-021-07313-3 PMC8723903

[39] Willis AW, Schootman M, Tran R, Kung N, Evanoff BA, Perlmutter JS, et al. Neurologist-associated reduction in PD-related hospitalizations and health care expenditures. Neurology. 2012;79(17):1774–80. doi: 10.1212/WNL.0b013e3182703f92 23054239 PMC3475618

[40] Pong RW, Pitblado JR. Geographic Distribution of Physicians in Canada: Beyond How Many and Where 2005 2021-12-09. Available from: https://secure.cihi.ca/free_products/Geographic_Distribution_of_Physicians_FINAL_e.pdf.

[41] Peacock D, Baumeister P, Monaghan A, Siever J, Yoneda J, Wile D. Perception of Healthcare Access and Utility of Telehealth Among Parkinson’s Disease Patients. Canadian Journal of Neurological Sciences. 2020:1–5. doi: 10.1017/cjn.2020.99 32450924

[42] Singh RL, Bush EJ, Hidecker MJC, Carrico CP, Sundin S. Considering Health Care Needs in a Rural Parkinson Disease Community. Progress in community health partnerships: research, education, and action. 2020;14(1):15–28. doi: 10.1353/cpr.2020.0005 32280120

[43] O’Gorman LD, Hogenbirk JC, Warry W. Clinical telemedicine utilization in Ontario over the Ontario telemedicine network. Telemedicine and e-Health. 2016;22(6):473–9. doi: 10.1089/tmj.2015.0166 26544163 PMC4892212

[44] Bronskill SE, Maclagan LC, Maxwell CJ, Iaboni A, Jaakkimainen RL, Marras C, et al., editors. Trends in Health Service Use for Canadian Adults With Dementia and Parkinson Disease During the First Wave of the COVID-19 Pandemic. JAMA Health Forum; 2022: American Medical Association. 10.1001/jamahealthforum.2021.4599 PMC8903126 35977228

[45] Starfield B, Shi L, Macinko J. Contribution of primary care to health systems and health. The milbank quarterly. 2005;83(3):457–502. 10.1111/j.1468-0009.2005.00409.x PMC2690145 16202000

[46] Muzerengi S, Herd C, Rick C, Clarke CE. A systematic review of interventions to reduce hospitalisation in Parkinson’s disease. Parkinsonism & related disorders. 2016;24:3–7. doi: 10.1016/j.parkreldis.2016.01.011 26803377

[47] Willis A, Schootman M, Evanoff B, Perlmutter J, Racette B. Neurologist care in Parkinson disease: a utilization, outcomes, and survival study. Neurology. 2011;77(9):851–7. doi: 10.1212/WNL.0b013e31822c9123 21832214 PMC3162639

[48] Shanmugasegaram S, Oh P, Reid RD, McCumber T, Grace SL. Cardiac rehabilitation barriers by rurality and socioeconomic status: a cross-sectional study. International journal for equity in health. 2013;12(1):72. doi: 10.1186/1475-9276-12-72 23985017 PMC3765803

[49] Yakerson A. Home care in Ontario: Perspectives on equity. International Journal of Health Services. 2019;49(2):260–72. doi: 10.1177/0020731418804403 30282497

[50] Forbes DA, Edge DS. Canadian home care policy and practice in rural and remote settings: Challenges and solutions. Journal of Agromedicine. 2009;14(2):119–24. doi: 10.1080/10599240902724135 19437267

