# Supplementary material for: Trends in health service use among persons with Parkinson’s disease by rurality: A population-based repeated cross-sectional study
Source: PLoS One. 2023 May 19;18(5):e0285585. doi: 10.1371/journal.pone.0285585 (PMC10198504; doi:10.1371/journal.pone.0285585)
Supplement: S1 Appendix — (DOCX) [file pone.0285585.s001.docx]

**APPENDIX**

**Supplementary Table 1. Description of Ontario health administrative databases**

| **Database** | **Description** |
| --- | --- |
| Registered Persons Database (RPDB) | The RPDB is a population-based registry maintained by the Ontario Ministry of Health, used to derive basic sociodemographic characteristics (age, sex, postal code) and date of death (for deceased individuals). The RPDB also indicates the time periods for which an individual was eligible to receive publicly funded health insurance benefits. |
| Canadian Institute for Health Information Discharge Abstract  Database (CIHI-DAD) | The CIHI-DAD is compiled by the Canadian Institute for Health Information (CIHI) and contains administrative, clinical (diagnoses and procedures/interventions), demographic, and administrative information for all admissions to acute care hospitals in Ontario. |
| National Ambulatory Care Reporting System (NACRS) | The NACRS database is compiled by the CIHI and contains administrative, clinical (diagnoses and procedures), demographic, and administrative information for all patient visits made to hospital- and community-based ambulatory care centres (emergency departments, day surgery units, hemodialysis units, and cancer care clinics) in Ontario. |
| Ontario Health Insurance Plan Claims Database (OHIP) | The OHIP claims database contains information on inpatient and outpatient services provided to Ontario residents eligible for the province’s publicly funded health insurance system by fee-for-service health care practitioners (primarily physicians) and “shadow billings” for those paid through non-fee-for-service payment plans. |
| Ontario Drug Benefit Database (ODB) | The ODB database contains prescription medication claims for those covered under the provincial drug program, including: all nursing home residents, those aged 65 years and older, patients receiving services under the Ontario Home Care program, those receiving social assistance, and residents eligible for specialized drug programs. |
| Continuing Care Reporting System Long-Term Care Database (CCRS-LTC) | The CCRS-LTC database is compiled by the CIHI and comprises all mandatory clinical assessments performed on nursing home residents in Ontario. |
| National Rehabilitation Reporting System (NRS) | The NRS database contains client data collected from participating adult inpatient rehabilitation facilities and programs across Canada. |
| Ontario Population Estimates and Projections (POP) | The POP database contain intercensal and post-censal estimates of the Ontario population by sex, age, and geographic areas. |
|  |  |
|  |  |

**Supplementary Table 2. Case ascertainment performance of health administrative data algorithms to identify persons with Parkinson’s Disease, using the electronic medical record (EMR) as the reference standard**

| **Algorithms** | **TP** | **TN** | **FN** | **FP** | **Sensitivity (95% CI)** | **Specificity**  **(95% CI)** | **PPV**  **(95% CI)** | **NPV**  **(95% CI)** | **Kappa** | **Youden Index** | **Prev**  **(%)** |
| --- | --- | --- | --- | --- | --- | --- | --- | --- | --- | --- | --- |
| ≥ 20 years |  |  |  |  |  |  |  |  |  |  |  |
| 2 P in 1 y | 152 | 72757 | 39 | 53 | 79.6 (73.9-85.3) | 99.9 (99.9-99.9) | 74.1 (68.2-80.1) | 99.9 (99.9-100.0) | 0.77 | 0.80 | 0.28 |
| **3 P in 2 y** | **138** | **72780** | **53** | **30** | **72.3 (65.9-78.6)** | **100.0 (99.9-100.0)** | **82.1 (76.4-87.9)** | **99.9 (99.9-99.9)** | **0.77** | **0.72** | **0.23** |
| (2 P in 1 y) or  (1 RX and (1 P) ± 6 months) | 167 | 72743 | 24 | 67 | 87.4 (82.7-92.1) | 99.9 (99.9-99.9) | 71.4 (65.6-77.2) | 100.0 (100.0-100.0) | 0.79 | 0.87 | 0.32 |
| 1 RX and (1 P) ± 6 months | 141 | 72775 | 50 | 35 | 73.8 (67.6-80.1) | 100.0 (99.9-100.0) | 80.1 (74.2-86.0) | 99.9 (99.9-100.0) | 0.77 | 0.74 | 0.24 |
| ≥ 65 years |  |  |  |  |  |  |  |  |  |  |  |
| 2 P in 1 y | 130 | 14158 | 32 | 39 | 80.2 (74.1-86.4) | 99.7 (99.6-99.8) | 76.9 (70.6-83.3) | 99.8 (99.7-99.9) | 0.78 | 0.80 | 1.20 |
| **3 P in 2 y** | **119** | **14175** | **43** | **22** | **73.5 (66.7-80.3)** | **99.8 (99.8-99.9)** | **84.4 (78.4-90.4)** | **99.7 (99.6-99.8)** | **0.78** | **0.73** | **0.98** |
| 1 RX and (1 P) ± 6 months | 134 | 14165 | 28 | 32 | 82.7 (76.9-88.5) | 99.8 (99.7-99.9) | 80.7 (74.7-86.7) | 99.8 (99.7-99.9) | 0.82 | 0.82 | 1.20 |

Note: Table shared by personal communication (Butt et al.)

Drugs only available to those ≥ 65 years. N = 73,001 (191 PD cases, EMR prevalence = 0.26%) ≥ 20 years. N = 14,359 (162 PD cases, EMR prevalence = 1.13%) ≥ 65 years.

All algorithms reflect a 30-day skip period between physician billing codes.

TP = True Positive; TN = True Negative; FN = False Negative; FP = False Positive; PPV = Positive Predictive Value; NPV = Negative Predictive Value; CI = Confidence Interval; Prev = Prevalence;

y = Years; P = Physician Billing Code; Rx = Prescription including levodopa drugs, MAO-B inhibitors, dopamine agonists and COMT inhibitor
